# Combined Effects of Temperature and Salinity on the Pharmacokinetics of Florfenicol in Nile Tilapia (*Oreochromis niloticus*) Reared in Brackish Water

**DOI:** 10.3389/fvets.2022.826586

**Published:** 2022-03-01

**Authors:** Tirawat Rairat, Yi-Kai Liu, Julia Chu-Nin Hsu, Chia-Yu Hsieh, Niti Chuchird, Chi-Chung Chou

**Affiliations:** ^1^Department of Fishery Biology, Faculty of Fisheries, Kasetsart University, Bangkok, Thailand; ^2^Department of Veterinary Medicine, College of Veterinary Medicine, National Chung Hsing University, Taichung, Taiwan; ^3^Department and Graduate Institute of Pharmacology, National Defense Medical Center, Taipei, Taiwan

**Keywords:** antimicrobials, aquaculture, environmental factor, fish, pharmacokinetics

## Abstract

Prudent antimicrobial use requires knowledge of pharmacokinetics (PK) in a specific fish species which in turn depends on water temperature and salinity. Although the influence of each individual factor is known, the combined effect is less clear. The objective of the current study was to investigate the effect of temperature and salinity concurrently on the PK of florfenicol (FF) in Nile tilapia reared in brackish water. Twenty-eight fish were divided into four groups and kept at one of two temperatures (24 vs. 32°C) and two salinity levels (5 vs. 15 ppt). The FF was administered at a single dose of 15 mg/kg body weight via oral gavage. The serum concentrations were analyzed by HPLC method and the PK parameters were analyzed by a 2-compartmental model. The result revealed that at 32°C, the elimination half-lives (t_1/2β_), time to reach the peak concentration (T_max_), area under the serum concentration-time curve (AUC), and mean residence time (MRT) were significantly decreased, while the clearance relative to bioavailability (CL/F) significantly increased compared to those at 24°C. The extents of these PK changes were similar at the two salinity levels. On the contrary, increasing the salinity from 5 to 15 ppt at a given temperature level produced no significant change in the PK behavior. Our finding indicated that only water temperature, but not salinity, is the major determinant factor governing the FF fate in the fish body.

## Introduction

Nile tilapia (*Oreochromis niloticus*) is among the fastest-growing species of tilapias, and has been popularly cultured worldwide. The global production of Nile tilapia from the aquaculture sector in 2018 was 4.5 million tons, being the fourth place after grass carp (*Ctenopharyngodon idella*, 5.7 million tons), Pacific white shrimp (*Litopenaeus vannamei*, 5.0 million tons), and silver carp (*Hypophthalmichthys molitrix*, 4.8 million tons) (1). The desirable characteristics of Nile tilapia for aquaculture production include fast growth (attaining a marketable size of 500–800 g within 6–8 months), good adaptability to captive conditions, tolerance to relatively poor water quality and crowding, relatively disease resistant, and feeding on low trophic levels (2, 3).

As a tropical freshwater fish, the optimal water temperature and salinity of Nile tilapia for the growth performance are between 28–32°C and 0–8 ppt, respectively (2, 4–7). Nevertheless, it has been suggested that Nile tilapia is also suitable for brackish water aquaculture for the salinity level up to 15 ppt (3, 8). The lower and upper lethal temperatures for Nile tilapia are 11–12°C and 42°C, respectively (2, 3), whereas the upper lethal salinity varies from about 20 ppt to about 40 ppt depending on the water temperature and the rate of salinity change (i.e., direct transfer vs. gradual acclimatization) (2, 9–11). The comparatively high performance in adaptability to a broad range of environmental conditions render Nile tilapia farming prosperous across different geographical locations, ranging from tropical to temperate climates, and from freshwater to brackish water.

The intensification of aquaculture has made industrial-scale food production systems possible. However, this benefit is often compromised by bacterial disease outbreaks, especially in overcrowded or poor management conditions. Among the most important bacterial pathogens of Nile tilapia are *Aeromonas hydrophila, Flavobacterium columnare, Streptococcus agalactiae, S. iniae*, and *Francisella orientalis* (synonym *F. asiatica*) (12, 13). While *A. hydrophila* and *F. columnare* diseases occur almost exclusively in freshwater-reared tilapia particularly at high temperature, infection with *S. agalactiae, S. iniae*, and *F. orientalis* can be found in both freshwater and saline water systems (12). Streptococcosis usually occurs at 32°C or warmer (13). Experimental challenge with *S. agalactiae* caused higher mortality at 33°C compared to 25°C in Nile tilapia (14), whereas the mortality due to *F. orientalis* experimental infection was more serious at 25°C than 30°C in both freshwater and marine water environments (15).

In the event of the bacterial epizootic, an antimicrobial drug often becomes the only effective measure to control the massive fish loss. In fact, the primary benefit of the use of veterinary medicines in aquaculture is that they support the development of intensive, industrial-scale aquatic farming, but the drug application is justifiable only for prudent and responsible use (16). Pharmacokinetics (PK) data are very crucial for the selection of the appropriate dose and dosing interval, and the prediction of the clinical outcome. Florfenicol (FF) is among the most popular antibacterial drugs approved for aquaculture use in many countries at the recommended dose of 10–15 mg/kg body weight (17–19). Despite the fact that many PK data of FF in fish species are available in the literature, most of these studies investigated the PK behavior at only one temperature and one salinity level. Nevertheless, the effect of water temperature on the PK of FF has been assessed in some fish species including Nile tilapia (20), common carp (*Cyprinus carpio*) (21), channel catfish (*Ictalurus punctatus*) (22), Japanese eel (*Anguilla japonica*) (23), crucian carp (*Carassius auratus*) (24), Wuchang bream (*Megalobrama amblycephala*) (25), and spotted halibut (*Verasper variegates*) (26), whereas the influence of salinity has been studied only in Nile tilapia (27). Unfortunately, to the best of the author's knowledge, the combined effect of temperature and salinity on the PK of FF has yet to be revealed in any fish species.

Regarding the PK characteristics of FF in Nile tilapia, the most important finding by our previous studies was that increasing either temperature (from 24 to 32°C, at 0 ppt) or salinity (from 0 to 15 ppt, at 28°C) resulted in faster FF elimination, thereby the larger dose of FF would be required at the warmer temperature or high salinity above 8 ppt (20, 27). The potential interaction between these two environmental factors, either additive or synergistic, might happen such that the fastest drug elimination would be seen at the warm saline water as opposed to the cool freshwater. The current study aimed to investigate the effect of temperature (24 vs. 32°C) and salinity (5 vs. 15 ppt) simultaneously on the PK behavior of FF in brackish water-reared Nile tilapia. The result would provide helpful information for antimicrobial chemotherapy with FF at different temperature and salinity levels.

## Materials and Methods

### Chemicals

FF and florfenicol amine (FFA) reference standard, sodium dodecyl sulfate, and ammonium hydroxide (NH_4_OH) were purchased from Sigma-Aldrich (St. Louis, MO). Acetonitrile (HPLC grade) and *N,N*-dimethylformamide were purchased from Avantor Performance Materials (Center Valley, PA). Triethylamine was purchased from Alfa Aesar, Thermo Fisher Scientific (Heysham, Lancashire, UK). Propylene glycol was purchased from AppliChem GmbH (Darmstadt, Germany). Sodium di-hydrogen phosphate anhydrous (NaH_2_PO_4_) was purchased from Panreac Química SLU (Barcelona, Spain). Phosphoric acid (H_3_PO_4_, 85% purity) was purchased from Scharlau (Barcelona, Spain).

### Experimental Fish

Nile tilapia (500–700 g), obtained from a commercial farm in Chiayi County, Taiwan, were acclimatized in a concrete pond containing freshwater at the College of Veterinary Medicine, National Chung Hsing University, Taiwan a few weeks before the experiment began. To study the simultaneous effects of water temperature (2 levels: 24 or 32°C) and salinity (2 levels: 5 or 15 ppt) on PK parameters of FF, a 2 × 2 factorial design was used. Twenty-eight fish were randomly assigned into one of the four groups (*n* = 7 for each group), namely, 24°C with 5 ppt, 24°C with 15 ppt, 32°C with 5 ppt, and 32°C with 15 ppt. Each fish was reared individually in a 70-L tank about 1 week before the drug administration. All fish were in good condition and showed no sign of stress during the acclimation period. The desired water temperature degrees were maintained by an aquarium heater in the air-conditioned room, while the predetermined salinities were adjusted by adding aquarium sea salt (Blue Treasure-Tropic Fish Sea Salt, Qingdao Sea-Salt Aquarium Technology, China) with adjusting rate of no greater than 5 ppt per day. The dissolved oxygen, pH, and total ammonia nitrogen throughout the study period were ≥5.0 ppm, 7.5–8.0, and <1.0 ppm, respectively. The animal study was approved by the Institutional Animal Care and Use Committee of National Chung Hsing University (IACUC approval No.: 108-134).

### Drug Administration and Blood Collection

The FF solution for oral gavage was prepared by dissolving the FF reference standard powder with 200 μL of *N,N*-dimethylformamide and adjusting the volume with 1,2-propylene glycol to attain the final concentration of 15 mg/mL. Each fish was administered the FF solution at a dose of 15 mg/kg body weight using a 1-mL syringe 8.4-cm stainless steel oral gavage tube. Approximately 0.4–0.5 mL of the blood sample was drawn from the caudal vessel without using anticoagulant at the predetermined time points, namely at 0.25, 0.5, 1, 2, 4, 8, 12, 24, 36, 48, 60, and 72 h post-administration. The fish were not fed after drug administration to avoid increased metabolic rate associated with food digestion and waste excretion, potentially helpful in minimizing stress during serial blood collection. The blood samples were allowed to clot at room temperature, and then centrifuged at 2000 × g for 10 min. The supernatants (serums) were collected and kept at −20°C until analysis.

### Sample Preparation and HPLC Analysis

The sample preparation and HPLC analysis of FF and FFA in the serum were modified from Xia et al. (28) and Wang et al. (29). The serum samples (200 μL) were extracted twice with 600 μL acetonitrile:ammonium hydroxide (98:2, v/v) and centrifuged at 2000 × g for 10 min. The supernatants were combined into a 50-mL tube and evaporated in the fume hood until completely dry. The residue was reconstituted with 200 μL mobile phase, and then filtered through 0.2-μm nylon syringe filter prior to the HPLC analysis.

**Figure 1:**
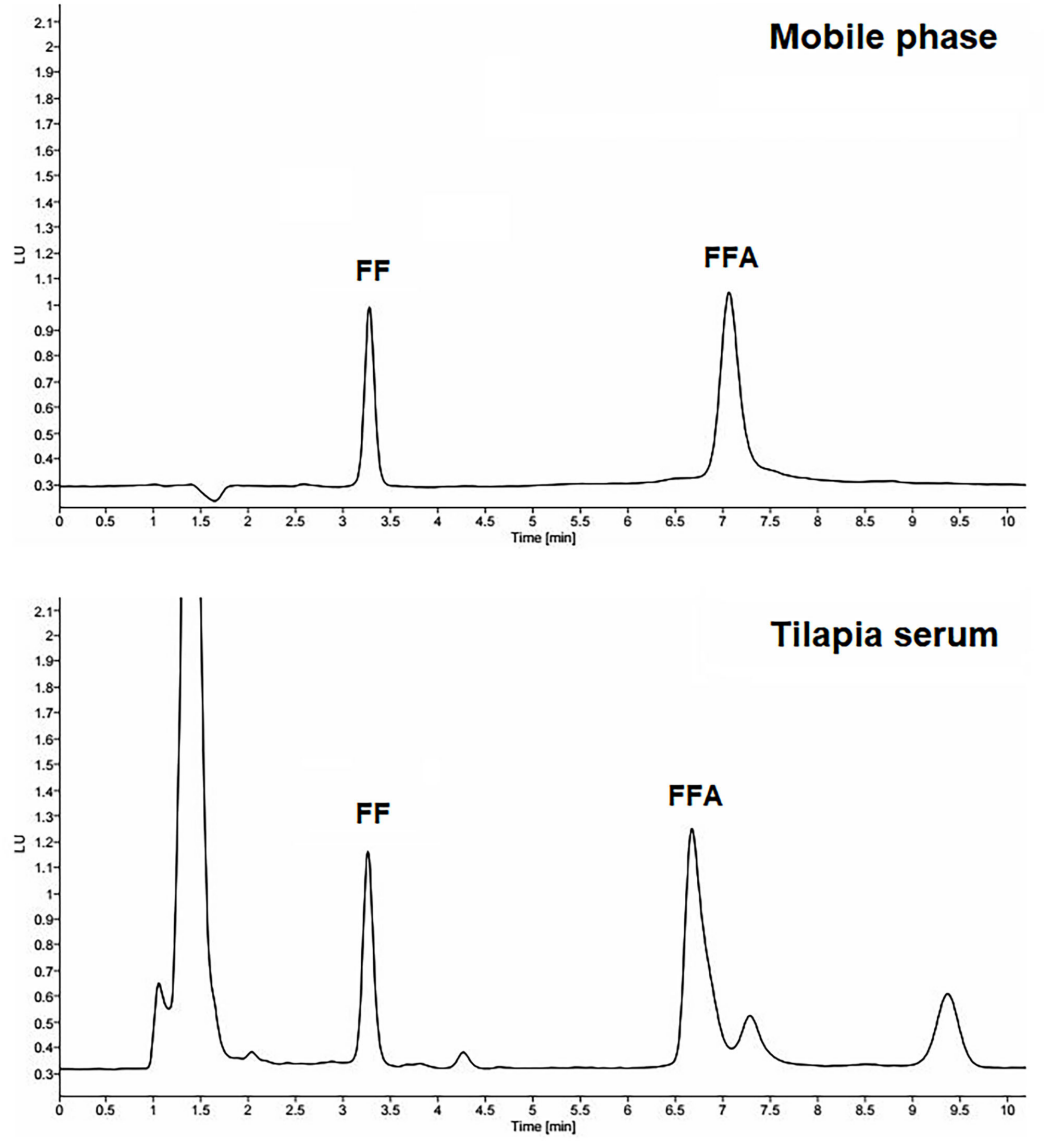
The representative HPLC chromatograms of 2 μg/mL reference standards of florfenicol (FF) and florfenicol amine (FFA) in the mobile phase and tilapia serum.

The concentrations of FF and FFA in serum were analyzed by the HPLC method. The mobile phase consisted of acetonitrile and phosphate buffer (a mixture of 10 mM NaH_2_PO_4_, 5 mM sodium dodecyl sulfate, 0.01% triethylamine, and adjusted pH to 4.8 by 85% H_3_PO_4_) at 35:65 v/v. The HPLC system consisted of a pump (1260 Infinity II, Agilent Technologies, Santa Clara, CA), fluorescence detector (G7121A, Agilent Technologies, Waldbronn, Germany), vialsampler (G7129A, Agilent Technologies, Waldbronn, Germany), and C-18 column with 5-μm particle size, 150 × 4.6 mm (Apollo, Hichrom, UK). The flow rate was 1 mL/min, and the excitation and emission wavelengths were 233 and 284 nm, respectively. The injection volume was 50 μL. The retention times of the FF and FFA peaks were about 3 and 7 min, respectively. The HPLC chromatograms of FF and FFA are presented in Figure 1.

To establish the matrix-matched calibration curves for quantification of FF and FFA concentration in the serum, the FF and FFA reference standards were spiked into the blank tilapia serum (collected from the same batch of unmedicated fish) to attain the final concentrations of 50, 100, 500 ng/mL, 1, 5, 10, and 20 μg/mL, then extracted and analyzed by the HPLC method described above (*n* = 5). The weighting factor of 1/x^2^ was applied.

### Pharmacokinetic Analysis

PK characteristics of FF were determined by the 2-compartmental model with a weighting scheme of 1/C. PKSolver 2.0 software (China Pharmaceutical University, Nanjing, China) (30) was used to analyze PK parameters including absorption rate constant (Ka), absorption half-life (t_1/2Ka_), distribution rate constant (α), distribution half-life (t_1/2α_), elimination rate constant (β), elimination half-life (t_1/2β_), transfer rate constant from the central (1) to the peripheral (2) compartment (k_12_), transfer rate constant from the peripheral (2) to the central (1) compartment (k_21_), elimination rate constant from the central compartment (k_10_), maximum serum concentration (C_max_), time to reach C_max_ (T_max_), area under the serum concentration-time curve (AUC), volume of distribution (Vd) of the central compartment relative to bioavailability (Vc/F), Vd during the elimination phase relative to bioavailability (Vz/F), Vd at steady-state relative to bioavailability (Vss/F), clearance relative to bioavailability (CL/F), and mean residence time (MRT).

### Statistical Analysis

The effects of temperature and salinity on PK parameters were simultaneously analyzed by two-way ANOVA (2 × 2 factorial design) using IBM SPSS Statistics version 27 software (IBM Corporation, Armonk, NY). When the assumption for a parametric statistical test was violated, the effects of temperature and salinity on a given PK parameter were separately compared by a nonparametric Mann-Whitney U test. In all cases, the *p*-value < 0.05 was considered statistically significant.

## Results

### HPLC Method Validations for Analysis of Florfenicol

The matrix calibration curves were linear over the range of 50 ng/mL to 20 μg/mL with a weighted r^2^ of 0.9982 (FF) and 0.9920 (FFA), the limits of detection (LOD) were 6 ng/mL (FF) and 12 ng/mL (FFA), the limits of quantification (LOQ) were 19 ng/mL (FF) and 35 ng/mL (FFA). The LOD and LOQ were calculated by 3.3^*^σ/S and 10^*^σ/S, respectively (σ = standard deviation of the y-intercept of the regression line; *S* = slope of the calibration curve). The percent extraction recovery was approximately 80–100% (FF) and 50–60% (FFA) at working ranges. The intra-day precision was <3% (for 0.1–20 μg/mL FF), 5.2% (for 50 ng/mL FF), <7% (for 0.1–20 μg/mL FFA), and 10.7% (for 50 ng/mL FFA). The inter-day precision was 7.4–10.5% (for 0.1–10 μg/mL FF) and 6.5–9.6% (for 0.1–10 μg/mL FFA). The accuracy was 91–99 % (FF) and 92–100 % (FFA).

### Pharmacokinetic Study

Although an attempt has been made to quantitate both FF and its major metabolite FFA simultaneously in the fish serum by the HPLC method, in general, the FFA was irregularly detected at low concentrations, often near or below the LOQ. Therefore, the FFA was excluded from the subsequent pharmacokinetic analysis.

The current study revealed that increasing the water temperature from 24 to 32°C caused significant changes in some key PK parameters of FF regardless of salinity levels (Table 1). Specifically, the t_1/2β_ were shortening from about 15 to 8 h, the T_max_ were shortening from about 1 to 0.6 h, the AUC were almost halved from about 400–200 h·μg/mL, the CL/F were increased from 0.04 to 0.06 L/kg/h, and the MRT were decreased from about 20 to 11 h. The t_1/2Ka_ and t_1/2α_ were also decreased as the temperature rose even though the results were statistically significant at only one salinity level (i.e., at 15 ppt for t_1/2Ka_ and at 5 ppt for t_1/2α_). In contrast, water temperature alteration produced no change in the C_max_ and all three Vd/F values (i.e., Vc/F, Vz/F, and Vss/F).

**Table 1:** Pharmacokinetic parameters (mean ± SD) of florfenicol following oral administration (15 mg/kg) at two temperatures and two salinities (*n* = 4 for the 32°C with 15 ppt group; *n* = 7 for the other three groups).

	**24**°**C**	**32**°**C**
**Ka (1/h): Absorption rate constant**		
5 ppt	1.78 ± 0.69^a, A^	2.42 ± 0.16^a, A^
15 ppt	1.51 ± 0.56^a, A^	3.39 ± 2.20^a, B^
**t**_**1/2Ka**_ **(h): Absorption half-life**		
5 ppt	0.39 ± 0.14^a, A^	0.29 ± 0.02^a, A^
15 ppt	0.46 ± 0.20^a, A^	0.20 ± 0.11^a, B^
**α** **(1/h): Distribution rate constant**		
5 ppt	1.25 ± 0.15^a, A^	2.13 ± 0.14^a, B^
15 ppt	1.20 ± 0.31^a, A^	1.73 ± 0.76^a, A^
**t**_**1/2α**_ **(h): Distribution half-life**		
5 ppt	0.56 ± 0.07^a, A^	0.32 ± 0.02^a, B^
15 ppt	0.58 ± 0.19^a, A^	0.40 ± 0.34^a, A^
**β** **(1/h): Elimination rate constant**		
5 ppt	0.048 ± 0.007^a, A^	0.083 ± 0.011^a, B^
15 ppt	0.045 ± 0.003^a, A^	0.086 ± 0.009^a, B^
**t**_****1/2**β**_ **(h): Elimination half-life**		
5 ppt	14.52 ± 1.95^a, A^	8.35 ± 0.09^a, B^
15 ppt	15.51 ± 1.16^a, A^	8.06 ± 0.82^a, B^
**k**_**12**_ **(1/h): Transfer rate constant from the central (1) to peripheral (2) compartment**		
5 ppt	0.72 ± 0.13^a, A^	1.25 ± 0.14^a, B^
15 ppt	0.63 ± 0.32^a, A^	0.94 ± 0.54^a, B^
**k**_**21**_ **(1/h): Transfer rate constant from the peripheral (2) to central (1) compartment**		
5 ppt	0.44 ± 0.09^a, A^	0.71 ± 0.06^a, B^
15 ppt	0.35 ± 0.11^a, A^	0.65 ± 0.16^a, B^
**k**_**10**_ **(1/h): Elimination rate constant from the central compartment**		
5 ppt	0.14 ± 0.01^a, A^	0.25 ± 0.04^a, B^
15 ppt	0.13 ± 0.04^a, A^	0.22 ± 0.08^a, B^
**C**_**max**_ **(μg/mL): Maximum serum concentration**		
5 ppt	29.59 ± 4.63^a, A^	27.75 ± 2.96^a, A^
15 ppt	25.66 ± 2.36^a, A^	27.97 ± 2.59^a, A^
**T**_**max**_ **(h): Time to reach C**_**max**_		
5 ppt	0.95 ± 0.20^a, A^	0.60 ± 0.04^a, B^
15 ppt	1.15 ± 0.33^a, A^	0.61 ± 0.14^a, B^
**AUC (h): Area under the serum concentration-time curve**		
5 ppt	423.28 ± 99.13^a, A^	233.00 ± 33.63^a, B^
15 ppt	393.40 ± 41.65^a, A^	235.66 ± 25.02^a, B^
**Vc/F (L/kg): Volume of distribution of the central compartment relative to bioavailability**		
5 ppt	0.27 ± 0.06^a, A^	0.27 ± 0.04^a, A^
15 ppt	0.32 ± 0.11^a, A^	0.32 ± 0.12^a, A^
**Vz/F (L/kg): Volume of distribution during the elimination phase relative to bioavailability**		
5 ppt	0.77 ± 0.11^a, A^	0.79 ± 0.11^a, A^
15 ppt	0.86 ± 0.09^a, A^	0.75 ± 0.03^a, A^
**Vss/F (L/kg): Volume of distribution at steady-state relative to bioavailability**		
5 ppt	0.71 ± 0.09^a, A^	0.73 ± 0.10^a, A^
15 ppt	0.79 ± 0.08^a, A^	0.69 ± 0.03^a, A^
**CL/F (L/kg/h): Clearance relative to bioavailability**		
5 ppt	0.037 ± 0.007^a, A^	0.066 ± 0.010^a, B^
15 ppt	0.039 ± 0.005^a, A^	0.064 ± 0.007^a, B^
**MRT (h): Mean residence time**		
5 ppt	20.34 ± 2.61^a, A^	11.70 ± 1.53^a, B^
15 ppt	21.35 ± 1.66^a, A^	11.19 ± 1.19^a, B^

*The means of half-lives are harmonic means whereas the means of the other PK parameters are arithmetic means. For each PK parameter, means with different small superscripts in each column and means with different capital superscripts in each row are significantly different from each other (p < 0.05)*.

**Figure 2:**
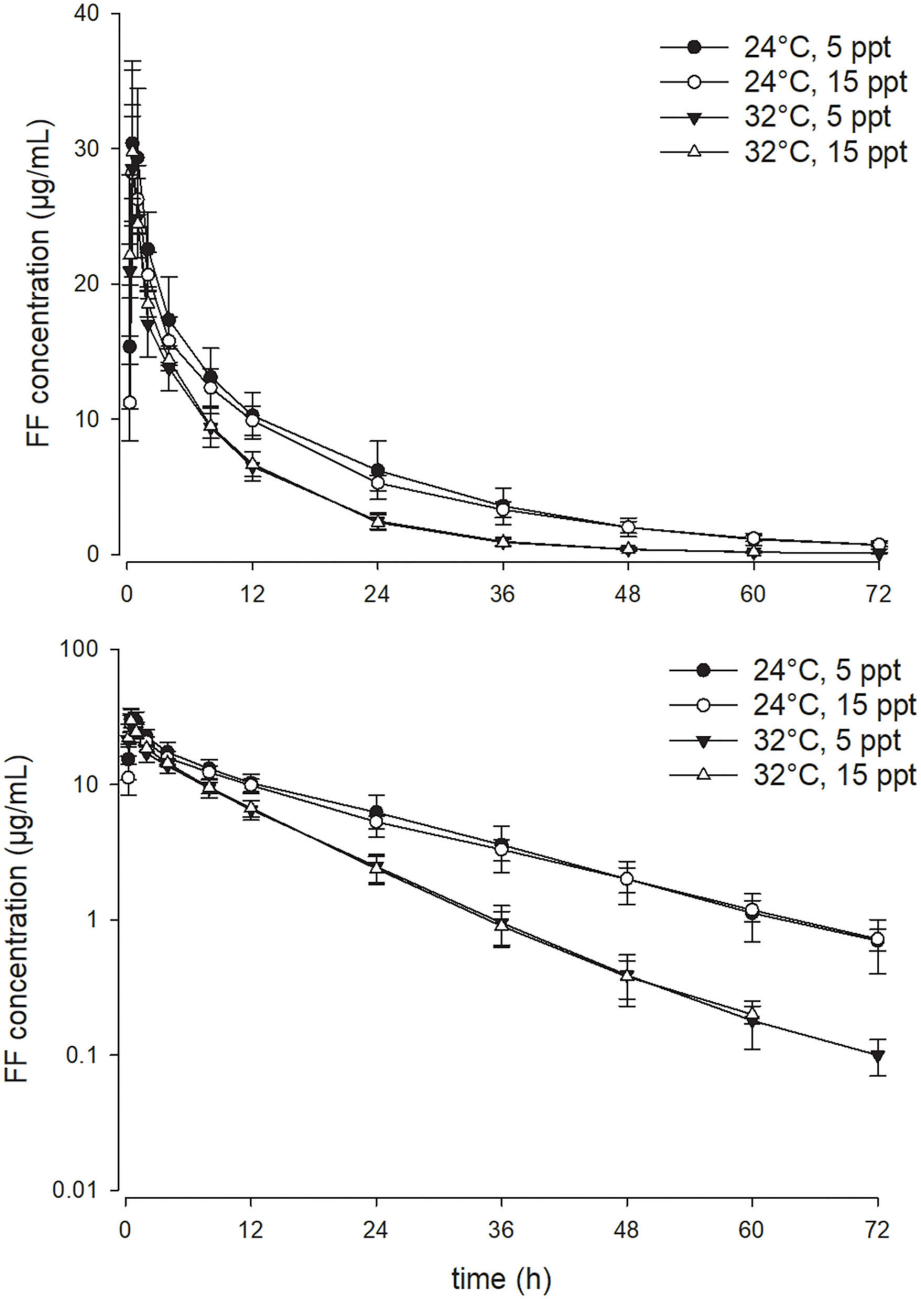
Linear (above) and semi-logarithmic plots (below) of the complete serum concentration-time profile from 0 to 72 h (mean ± SD) of 15 mg/kg florfenicol following oral (PO) administration at two temperatures and two salinities (*n* = 4 for the 32°C with 15 ppt group; *n* = 7 for the other three groups).

**Figure 3:**
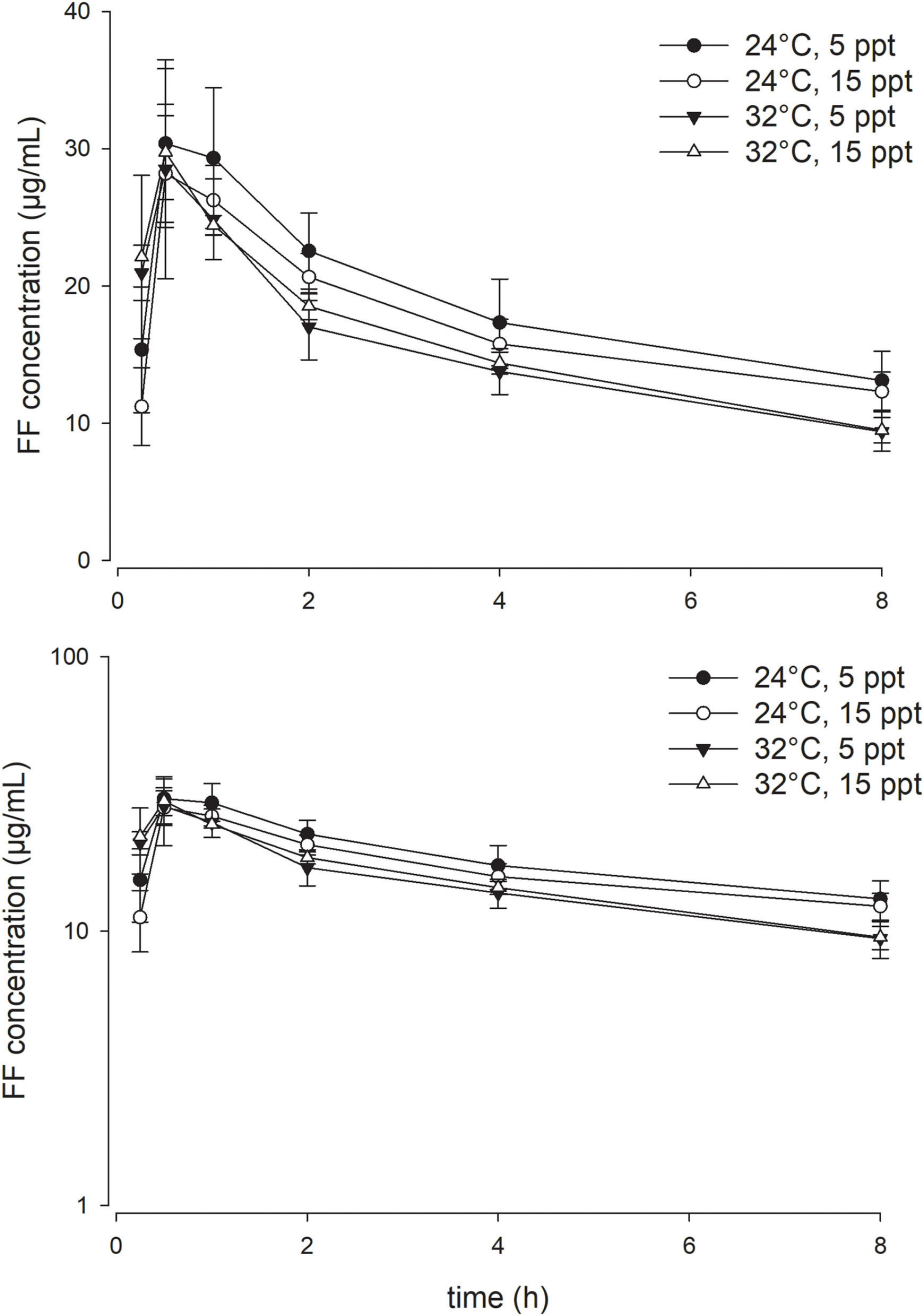
Linear (above) and semi-logarithmic plots (below) of serum concentration-time profile during the first 8 h (mean ± SD) of 15 mg/kg florfenicol following oral (PO) administration at two temperatures and two salinities (*n* = 4 for the 32°C with 15 ppt group; *n* = 7 for the other three groups).

As opposed to the effect of the temperature, enhancing water salinity from 5 to 15 ppt exerted no significant impact on the PK parameters of FF in the current experimental setting. At a given water temperature degree, the serum concentration-time profiles of FF at the two salinities were almost superimposed on each other (Figures 2, 3). Throughout the study period, all fish survived the experiment except three fish in the 32°C with 15 ppt group, rendering the sample size of that group with four fish.

## Discussion

Even though the water temperature of 28–32°C and salinity of 0-8 ppt are generally considered optimal for Nile tilapia culture (2, 4–7), the fish is also well recognized for its adaptability to wide environmental conditions. In a relatively stress-free rearing environment, at least some strains of Nile tilapia can tolerate a wide range of water temperature (16–37°C) and salinity (0–22 ppt) with 98–100% survival rates (5, 31), while the other strains suffered certain degrees of mortality when the salinity was increased above 8 ppt, irrespective of the water temperature (4). In the current experimental conditions, all individual fish in the 24°C with 5 ppt, 24°C with 15 ppt, and 32°C with 5 ppt groups were successfully raised toward the end of the predetermined sampling time point. For the Nile tilapia in the 32°C with 15 ppt group, however, 3 out of the 7 fish died before the experiment finished which indicated that the combination of high temperature and high salinity was stressful and deemed unsuitable for Nile tilapia culture in some circumstances (such as high stocking density) even if it has been suggested that Nile tilapia can be successfully reared at 32°C and salinity up to 15 ppt (2, 3, 8). In addition, Nile tilapia cultured at a very low temperature (14°C) also was less tolerant to high salinity water compared to those reared within the optimal temperature (30°C) (10). Thus, at the extreme ends of their optimal temperature and salinity ranges, Nile tilapia is more susceptible to stress-induced mortality. This finding might imply the existence of interplay between the water temperature and salinity on certain physiological aspects. Nevertheless, this interaction did not affect the PK results of FF in the current experimental setting (as discussed below). Fish farmers who raise Nile tilapia in brackish water above 8–10 ppt in a tropical climate should pay attention to avoid any potential stressors and monitor the fish health closely particularly during the hot summer.

In the current experimental setting, certain PK parameters including t_1/2Ka_, t_1/2β_, T_max_, AUC, CL/F, and MRT of FF were significantly changed when the temperature was increased from 24 to 32°C, whereas increasing the salinity from 5 to 15 ppt produced no significant effect in any PK parameters investigated. The result suggested that the temperature has a greater impact on the absorption, distribution, and elimination processes of FF compared to the salinity. It is well recognized that water temperature has a strong influence on fish metabolic rate (32, 33) and other physiological functions such as cardiac output (34, 35). Likewise, the activities of xenobiotic-metabolizing enzymes are usually higher at the warmer temperature (36–40). The changes in enzyme activities were attributed to the different isozymes produced at different temperature levels rather than the alteration of the enzyme content *per se* (36–38). Therefore, the more rapid FF elimination at 32°C in the present study was probably attributed to the temperature-induced enhancement of FF-metabolizing enzyme activity. Even though the identity of the enzyme has not been revealed yet, based on literature reviews it likely belongs to a member of the CYP3A subfamily (41–43). On the contrary, the faster FF absorption at higher temperature was likely a result from the increased gut blood flow mainly due to the increased cardiac output (44, 45).

The findings that increasing water temperature leads to a decrease in AUC, t_1/2Ka_, t_1/2β_, T_max_, and MRT, and an increase in CL/F of FF in Nile tilapia reared in 5 and 15 ppt salinities were generally in agreement with our previous study with freshwater-reared Nile tilapia using a similar experimental design (20), indicating that the salinity has little, if any, effect on the PK of FF in Nile tilapia. Nevertheless, some minor disagreements between the two studies were noticed. For instance, while the C_max_ and Vd/F of the brackish water-reared Nile tilapia in the current study were not significantly affected by the temperature changes, the opposite was true for the Nile tilapia cultured in 0 ppt water (20). Other than the possible effect of salinity difference, batch-to-batch variation of Nile tilapia strains between the two experiments may be another possible explanation since different tilapia strains could have different growth performance and adaptability in saline water (46–48). It is worth mentioning that the directions of change for the AUC, t_1/2β_, T_max_, and MRT (which decrease at a higher temperature) and CL/F (which increase at a higher temperature) of FF were almost consistently observed across various fish species and experimental conditions (20–26). In contrast, the effects of water temperature on C_max_ and Vd/F were more elusive. For example, increasing temperature resulted in decreased C_max_ and increased Vd/F in Nile tilapia (20), increased C_max_ and decreased Vd/F in crucian carp (24) and Japanese eel (23), or no change in both parameters in channel catfish (22). Other than FF, the variability in the change direction of C_max_ and Vd/F following temperature change was seen in other antibacterial drugs as well (49–52).

The potential interaction between the temperature and salinity factors on PK was not evident in the current study. When the water temperature was raised from 24 to 32°C, the extents of the changes in the AUC, t_1/2β_, CL/F, and MRT were similar, being about 1.7–1.9-fold differences, at both salinity levels. The apparent lack of salinity effect was somewhat unexpected since our previous work demonstrated significant faster FF elimination in Nile tilapia reared at higher salinity (>8 ppt) at 28°C (27), and other studies with oxytetracycline in tilapia (*Oreochromis* sp.) (53), oxolinic acid in rainbow trout (54, 55), and flumequine in Atlantic salmon (56, 57) also supported this finding. Nonetheless, the differences in salinity levels in these previous studies were large, namely from 0 ppt to about 30 ppt in the cases of oxytetracycline in tilapia and quinolones in salmonids (54–57) or from 0 to 15 ppt in the case of FF in Nile tilapia (27). On the other hand, the 10 ppt-salinity difference (from 5 to 15 ppt) in the present study might not be big enough to significantly affect the fish physiological functions and reveal statistically significant differences.

The stronger influence of the water temperature over the salinity on the FF elimination was foreseeable. While the salinity is more important than temperature in influencing the plasma/serum osmolality and gill Na^+^/K^+^-ATPase activity, the effect of water temperature is stronger in affecting the growth rate and feed efficiency of Nile tilapia (5, 31). As our previous work found that Nile tilapia can maintain the serum osmolality at a relatively constant level, around 322–347 mOsm/kg over the salinity range of 0–15 ppt, such that the osmolality is unlikely to play any significant role in a drug elimination mechanism (27). However, other salinity-induced physiological changes such as salt excretion or water retention cannot be ruled out and are worth further investigation, even though the salinity effect on FF PK may not be discernable in our study. In contrast, a higher metabolic rate at a warmer temperature not only directly affects the growth, feed utilization, and digestive enzyme activities (5, 31, 58), but also drug-metabolizing enzyme activities (39, 40). For that reason, water temperature is considered the principal environmental factor determining the rate of drug elimination from the fish body.

The results from the present study expanded our previous knowledge and provided a more complete picture of the effects of temperature and salinity on the PK behavior of FF in Nile tilapia. Temperature significantly affects certain PK parameters of FF, especially those in association with the drug elimination in both freshwater (20) and brackish water-reared Nile tilapia. Little interaction between the temperature and salinity was observed in the current setting. In addition, a previous finding that salinity effect is less important than temperature holds true not only at 28°C (27) but is also applicable over the entire range of the preferred temperature for Nile tilapia aquaculture (24–32°C).

## Conclusion

Temperature, but not salinity, dictated the pharmacokinetic behavior of FF in brackish water-reared Nile tilapia. Following the increment of water temperature from 24 to 32°C at both salinities (5 and 15 ppt), the t_1/2β_, T_max_, AUC, and MRT were decreased almost twofold while the CL/F increased at a similar extent. The current finding suggested that the PK parameters determined at a low salinity level can practically be applied to the medium salinity level as well and vice versa.

## Data Availability Statement

The original contributions presented in the study are included in the article/supplementary material, further inquiries can be directed to the corresponding author.

## Ethics Statement

The animal study was reviewed and approved by Institutional Animal Care and Use Committee of National Chung Hsing University (IACUC Approval No.: 108-134).

## Author Contributions

TR performed the PK analysis, data interpretation, and drafted the manuscript. Y-KL and JC-NH performed the animal experiment and HPLC analysis. C-YH and NC reviewed and edited the manuscript. C-CC conceived and supervised the project. All authors read and approved the final manuscript.

## Funding

This research was funded by the Ministry of Science and Technology, Taiwan, Grant Number: MOST 109-2313-B-005-015-MY3.

## Conflict of Interest

The authors declare that the research was conducted in the absence of any commercial or financial relationships that could be construed as a potential conflict of interest.

## Publisher's Note

All claims expressed in this article are solely those of the authors and do not necessarily represent those of their affiliated organizations, or those of the publisher, the editors and the reviewers. Any product that may be evaluated in this article, or claim that may be made by its manufacturer, is not guaranteed or endorsed by the publisher.
